# Alarming Increase of Ketoacidosis Prevalence at Type 1 Diabetes-Onset in Austria—Results From a Nationwide Registry

**DOI:** 10.3389/fped.2022.820156

**Published:** 2022-02-14

**Authors:** Katrin Nagl, Thomas Waldhör, Sabine E. Hofer, Maria Fritsch, Dagmar Meraner, Christine Prchla, Birgit Rami-Merhar, Elke Fröhlich-Reiterer

**Affiliations:** ^1^Department for Pediatrics and Adolescent Medicine, Medical University of Vienna, Vienna, Austria; ^2^Department for Epidemiology, Center of Public Health, Medical University of Vienna, Vienna, Austria; ^3^Department for Pediatrics, Medical University of Innsbruck, Innsbruck, Austria; ^4^Department of Pediatrics and Adolescent Medicine, Medical University of Graz, Graz, Austria; ^5^Department for Pediatrics, Clinic Donaustadt, Vienna, Austria

**Keywords:** diabetes, ketoacidosis, population-based registry, COVID-19, general pediatrics

## Abstract

**Objective:**

We analyzed the annual prevalence of onset-DKA (diabetic ketoacidosis) from 2012 to 2020 with a sub-analysis for lockdown-periods during the COVID-19 pandemic in 2020.

**Design:**

All newly diagnosed children with type 1 diabetes (T1D) aged <15 years are prospectively registered in the population-based Austrian Diabetes Incidence Study in Austria.

**Main Outcome Measures:**

The annual DKA prevalence was analyzed using Joinpoint regression. Definition of DKA: pH <7.3, mild DKA: pH 7.3 to ≤ 7.1, severe DKA: pH <7.1. DKA prevalence during the lockdown periods in 2020 and the corresponding periods in 2015–2019 were examined using Fisher's exact test.

**Results:**

In the years 2012–2020 the mean prevalence for onset-DKA in Austria was 43.6% [95%CI (confidence interval): 41.6, 45.7] and thus above the mean prevalence of previous decades (1989–2011) of 37,1 % (95%CI: 35.6, 38.6). A particularly high prevalence was found among children <2 years of age (72.0% DKA, 32.8% severe DKA). No significant gender difference was found. Prevalence of severe DKA at T1D-onset increased significantly since 2015 (*p* = 0.023). During the lockdown in 2020, 59.3% of children were diagnosed with DKA at T1D-onset, compared to 42.1% during the previous 5 years (*p* = 0.022). Moreover, 20% of children had severe DKA at T1D diagnosis, compared to 14% during the comparison period.

**Conclusions:**

The previously already high prevalence of DKA at T1D-onset has further increased over time. The COVID-19 pandemic has exacerbated the problem of a late or delayed diagnosis of diabetes in children resulting in onset-DKA. The alarmingly increased prevalence of DKA in Austrian children with T1D calls for urgent action.

## What's Known on This Subject

- For decades the rate of diabetic ketoacidosis (DKA) at onset of type 1 diabetes (T1D) in children in Austria has remained high but stable.
- Past large awareness campaigns were not able to reduce the high prevalence of onset-DKA in Austria.

## What This Study Adds

- Years before the COVID-19 pandemic, a rapid increase of prevalence and severity of onset-DKA has been documented in Austria's population-based Diabetes Incidence Registry.
- This preexisting trend was aggravated by the lockdown measures during the COVID-19 pandemic of 2020.

## Introduction

When a child presents with diabetic ketoacidosis (DKA) at diagnosis of type 1 diabetes (T1D), there usually already has been a prior history of several weeks with classic diabetes-related symptoms (1, 2). DKA can lead to life-threatening complications (3), persistent neurological damage (4, 5), and is independently related to worse long time glucose control (6). Its prevalence at T1D onset varies largely between countries, the overall adjusted DKA prevalence between 2006 and 2016 from 13 higher-income countries was 29.9% (7). For decades (1989–2011), the rate of diabetic ketoacidosis (DKA) at onset of type 1 diabetes (T1D) in children in Austria has remained stable at a high level of around 37% (8, 9), despite a significant increase in T1D incidence during the same time period (9) and a nation-wide, community-based poster-campaign for the information about diabetes-specific symptoms and prevention of onset-DKA in T1D in 2009 (8).

Data from the neighboring country Germany, more precisely the German Diabetes Follow-up Registry (DPV) always showed lower (7), but slightly increasing rates of onset DKA in the last years, but a significant increase of onset-DKA during the first 2 month of the COVID pandemic in 2020 compared to the prior 2 years (10). In addition, the COVID pandemic did not have any short-term effects on the T1D incidence in Germany (11).

In the present study we analyzed the annual rates of onset DKA from 2012 to 2020 with a sub-analysis for the lockdown-period during the first phase of the COVID-19 pandemic.

## Methods

### Austrian Diabetes Incidence Study

Since 1989, the Austrian Diabetes Incidence Study Group has been collecting data of all newly diagnosed children with diabetes (up to the age of 15 years). All new diabetes onsets are prospectively registered by a network of all Austrian pediatric hospitals and diabetologists. As described in previous studies (8, 12), the following data have been collected at diagnosis of T1D: age, sex, date of diagnosis, date of first insulin, clinical symptoms of diabetic ketoacidosis (DKA) at onset (hyperventilation, unconsciousness), height, weight, laboratory data at onset (glucose, pH, HCO3, ketones, HbA1c, and diabetes-specific antibodies), and family history of diabetes. As described earlier (12, 13), completeness of ascertainment was >93% using the capture-recapture method has been used, as recommended by the WHO DiaMond Project (14).

DKA was defined as a pH <7.3, mild DKA as pH ≤ 7.3 to ≤ 7.1 and severe DKA as pH <7.1. Data between January 1, 1989, and December 31, 2020, were available. For this paper the latest data from 2012 to 2020 were analyzed and compared to previous data already published (1989–2011) from the Austrian Diabetes Incidence registry (8, 9).

### COVID-19 Pandemic and Lockdown 2020

The effects of the COVID-19 pandemic in Austria resulted in three hard lockdown periods during 2020: March 16th–May 15th, November 17th–December 7th, and December 26th 2020–February 7th 2021 with many restrictions and limited access to medical care (15). Detailed information on specific lockdown measures is given in the supplement.

### Statistical Analysis

The annual rates plus 95% confidence intervals of DKA, mild DKA and severe DKA at T1D onset between 2012 and 2020 in this population-based study were calculated in total and separately for the age groups 2 to <5, 5 to <10, and 10 to <15 years. Time trend of age-standardized DKA rates for any DKA, mild and severe DKA at onset as well as annual percent change and *p*-values were estimated by Joinpoint analysis (16). Slopes of trends modeled by Joinpoint analysis are described by annual percent change (APC) and 95% confidence intervals.

A logistic regression model was calculated to describe the effects of age at T1D onset (continuous variable), year of diagnosis (2012–2020), and gender on the probability for presentation with DKA at T1D onset. The effect of age at T1D onset was modeled by a cubic B-spline to allow for a non-monotonic effect.

To analyze the effects of the lockdown periods on the proportion of onset DKA during the COVID-19 pandemic in 2020, the proportion of onset DKA during lockdown in 2020 was compared with the aggregated corresponding comparison periods from 2015 to 2019, namely: March 16th–May 15th, November 17th–December 7th, and December 26th–December 31st. This was done to exclude potential seasonal effects. A 2-sided Fisher's Exact Test was used for comparison. The period of comparison was not extended beyond 2015–2019 to exclude any long-time varying effects.

For all analyses a *p*-value below 0.05 was considered statistically significant. No adjustment for multiple tests was done and *p*-values are to be interpreted as exploratorily only. Figures and all other calculations were done in SAS version 9.4 (SAS Institute Inc., Cary, NC, USA).

## Results

### DKA at T1D Onset From 2012 to 2020

In the years 2012–2020, the prevalence of onset-DKA in Austria was 43.6% in total, a third of which fulfilled criteria of severe DKA (Table 1).

**Table 1:** Prevalence (%) of DKA and age at T1D onset in children in Austria between 2012 and 2020.

	**Age at T1D onset (years)**				
DKA	0 to <2	2 to <5	5 to <10	10 to <15	Total
No DKA (pH ≥ 7.3)	35 (28.0)	233 (57.5)	511 (61.1)	563 (55.5)	1,342 (56.4)
Mild DKA (pH <7.3 and ≥ 7.1)	49 (39.2)	117 (28.9)	225 (26.9)	306 (30.2)	697 (29.3)
Severe DKA (pH <7.1)	41 (32.8)	55 (13.6)	100 (12.0)	145 (14.3)	341 (14.3)
Total	125	405	836	1,014	2,380 (100)

*Data are number of children and percentages.*

*DKA, diabetic ketoacidosis; T1D, type 1 diabetes*.

A particularly high prevalence of onset-DKA was documented among children under 2 years of age with more than 72%. Almost half of children under 2 years of age were admitted with severe DKA at T1D onset. Comparable DKA proportions were found in the age groups 2 to <5, 5 to <10, and 10 to <15 years, however with a slightly higher onset-DKA proportion in the oldest age group (Table 1). We did not observe any cases of death at the onset of T1D.

### Temporal Trends—Joinpoint Analysis

**Figure 1:**
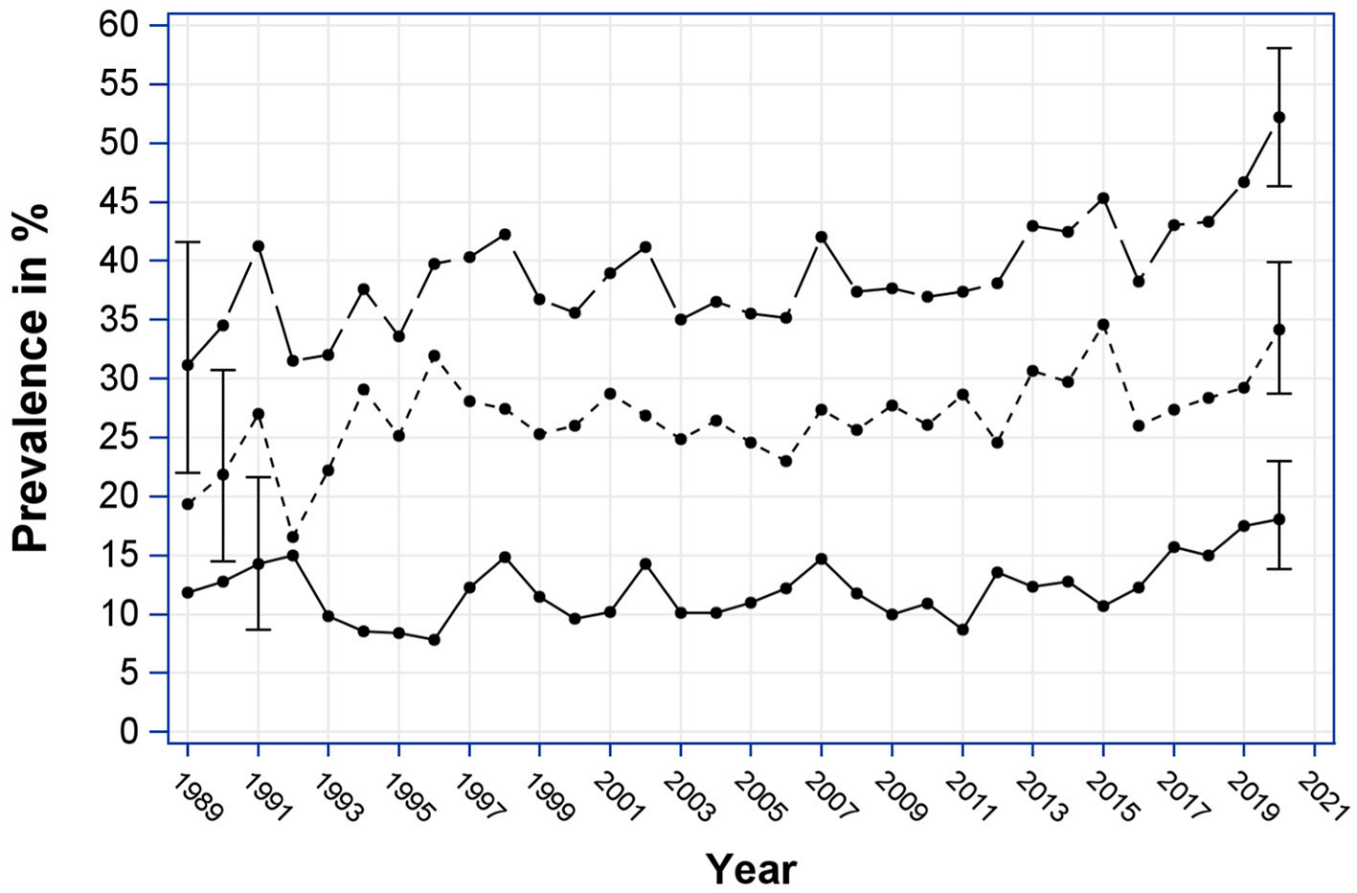
Annual prevalence of onset-DKA (%) from 1989 to 2020, by severity with 95% confidence intervals. Long dashed, DKA; short dashed, mild DKA; solid, severe DKA.

As can be seen in Figure 1, the prevalence of DKA at T1D-onset among children in Austria has increased over time. Joinpoint analysis for the period 1989–2020 revealed a significant steady increase of onset-DKA with an APC of 0.59% (*p* = 0.005) until the year 2017, where a significant joinpoint was found (*p* < 0.05). From 2017 onwards, there was a strong increase in the annual DKA rate, with an APC of 7.69%, which, however, was not statistically significant due to the short observation period of further 4 years only.

### Temporal Trends—Joinpoint Analysis—Mild and Severe DKA

Analyzing temporal trends of annual DKA according to the severity of DKA revealed the following results:

For mild DKA a continuous significant increase in DKA prevalence between 1989 and 2020 with an APC of 0.78% (*p* = 0.002) was observed. Prevalence of severe DKA did not change significantly between 1989 and 2015. In 2015, a significant Joinpoint (*p* < 0.05) was found followed by a significant increase in prevalence with an APC of 9.78 (*p* = 0.023).

### Effect of Year of Diagnosis, Age, and Gender on Probability for Onset-DKA

**Figure 2:**
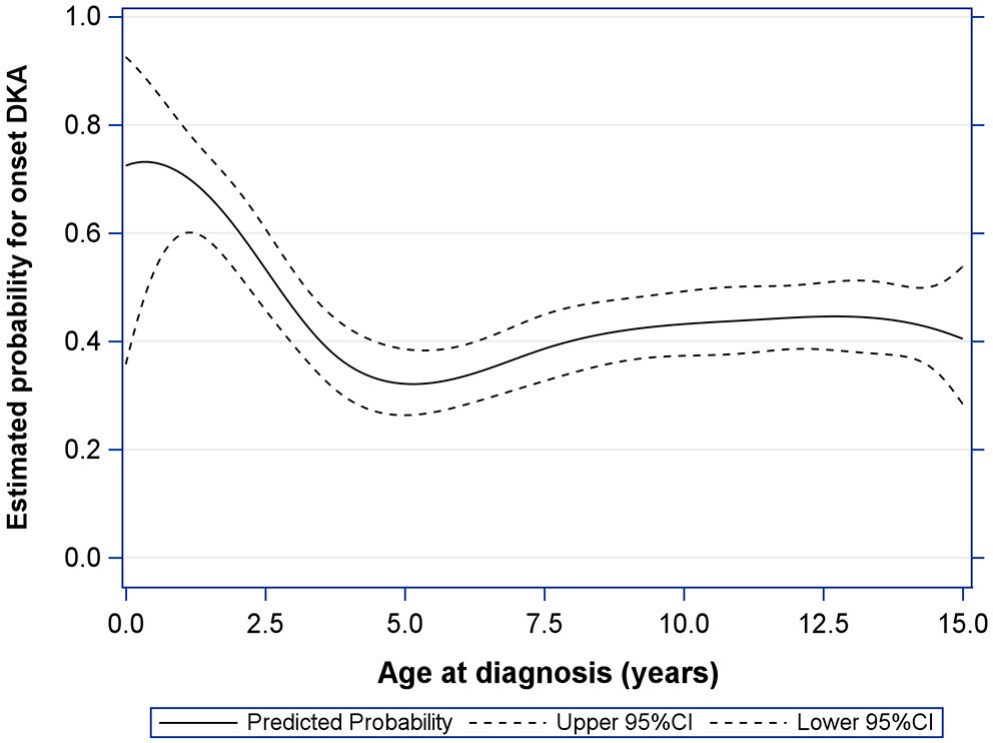
Effect of age at diagnosis on estimated probability for DKA at T1D-onset (with upper and lower 95% confidence limit), adjusted for gender and year of diagnosis.

A logistic regression model confirmed the significant relationship (*p* = 0.027) with the year of diagnosis and showed a continuous increase in the probability of a DKA at T1D onset from 2012 to 2020. The relationship with age at diagnosis was shown to be highly significant (*p* < 0.001) with a very high probability of onset DKA in toddler age, a lower risk in school entry age and, in turn, a slightly increasing risk in adolescence (Figure 2). Adjusted for year of diagnosis, age at onset, there was no significant gender influence on the occurrence of onset DKA.

### Comparison of Lockdown to Corresponding Timespan

During the lockdown period caused by the COVID-19 pandemic in 2020, we observed a significant increase of 17.2% in onset-DKA prevalence of children (*p* = 0.022, Table 2) from 42.1% during non-lockdown time periods from the 5 previous years to 59.3% during lockdown periods. Twenty percentage of children had severe DKA at T1D diagnosis, compared to 14% during the comparison period.

**Table 2:** Prevalence of DKA (%) at T1D onset in children in Austria during the COVID-19 related lockdown periods in 2020 in comparison to the corresponding previous 5-year periods (2015–2019).

	**Year of diagnosis**		**p-value**
	**2015–2019**	**2020 lockdown**	
No DKA (pH ≥ 7.3)	175 (58.0)	24 (40.7)	0.022
Any DKA (pH <7.3)	127 (42.1)	35 (59.3)	
Mild DKA (pH <7.3 and ≥7.1)	84 (27.8)	23 (39.0)	
Severe DKA (pH <7.1)	43 (14.2)	12 (20.3)	
Total	302	59	

*Data are number of children and percentages.*

*DKA, diabetic ketoacidosis; T1D, type 1 diabetes; COVID-19, corona-virus disease*.

## Discussion

In the years 2012–2020, the mean prevalence for onset-DKA in Austria was 43.6% and thus above the mean level of previous decades (1989–2011) of 37.2% (8). In comparison with other European countries (7), the Austrian onset-DKA prevalence had already been on a high level, albeit stable for decades (8, 9). Our new results are particularly worrying because of a steep increase in the prevalence of DKA at T1D diagnosis already years before the COVID-19 pandemic. Other countries, such as Australia, Germany, and the USA also reported a slight increase in onset DKA prevalence (7, 17), while Italy, also a neighboring country even reported a significant decrease in onset-DKA (7).

The increase in onset-DKA is unlikely to be linked to the incidence of T1D in children in Austria, as the population-based Austrian diabetes incidence study of late reported (12) a recent slowing down of the steep increase of T1D incidence of previous years. This development was attributed to the decline in the T1D incidence in small children (12).

### Reasons for a High Onset-DKA Prevalence in Austria

Young children and people from ethnic minorities in particular have the highest risk of onset DKA (7, 18). In line with this, our data also show dramatically high rates of DKA and severe DKA in young children, which again indicates more aggressive disease progress or vulnerability to dehydration in this age group (9).

Many children and their relatives report ongoing diabetic symptoms weeks or even months before the diagnosis of T1D. Data from the SWEDIAB study show that more than 40% of children who had been in contact with primary health care providers due to clearly diabetes-related symptoms before their final hospitalization had a delayed referral. Children with a delayed referral had significantly lower pH-values at hospital admission (19). Time is often wasted on pre-referral investigations (20). Point-of-care testing of, for example, blood or urine glucose by the primary health care provider facilitates early tertiary referral and can thus prevent the occurrence of DKA (20).

In general, in Austria, the access to health care with almost 100% coverage is available to everyone 24/7 based on state-organized health insurance systems. However, there has been a structural shortage of pediatricians in recent years (21). Families therefore often seek the help of general practitioners (GP) who, however, might only have limited pediatric expertise (8). Nonetheless, due to the relative rarity of T1D also trained pediatricians sometimes do not diagnose the disease timely.

No statistically significant association with the occurrence of onset DKA was found for gender adjusted for age at onset and year of diagnosis. This fits in with the results of a multinational study comparing temporal trends of onset-DKA (7). Only three out of 13 different countries reported a higher risk for onset-DKA in girls, while data from Wales even showed a higher prevalence of onset-DKA in boys. In most participating centers, no significant gender difference was found (7).

### Onset DKA During the COVID-19 Pandemic

The current COVID-19 pandemic and the measures to combat the pandemic have exacerbated many pre-existing problems (22). Our data show a dramatic increase in the already high onset DKA prevalence, as well as a significant increase in the severity of the presentation.

Many smaller studies already showed a significantly worsened presentation of children at T1D diagnosis during the first wave of the COVID-19 pandemic (23–28).

An analysis of the Finnish Pediatric Diabetes Registry showed that the increase in the prevalence and severity of onset-DKA was not due to infections with SARS-CoV-2, but mainly to delayed diagnosis due to limited healthcare accessibility and changed parental behavior, mainly driven by fear of COVID-19 (29–31).

An analysis of the German Diabetes Registry DPV also showed a significant increase in the frequency of onset DKA during the first lockdown period. Again, children <6 years of age and children with migration background had the highest risk of onset DKA (32). What can probably be ruled out from the current perspective is that the COVID-19 pandemic led to an increase in the incidence of T1D (11).

### Measures to Reduce the Frequency of Onset-DKA

Regarding the possible lethality of diabetic ketoacidosis (3), the further course of the diabetes disease (33, 34), and the fact that diabetic ketoacidosis can cause permanent neurological damage (4, 5), it is important to react to the critical trend of recent years and initiate measures.

In 2009, a comprehensive information and DKA prevention campaign to increase diabetes awareness was carried out nationwide in Austria—unfortunately without success (8). In the following years, albeit at regional level in Styria, which is home to 14% of the Austrian population, another poster and information campaign was carried out locally and, again without success.

However, similar campaigns were quite successful in other countries. The Stuttgart Ketoacidosis Awareness Campaign, in which families were informed about typical diabetic symptoms during pre-school health examinations, led to a reduction in onset DKA prevalence from 28 to 16% (35). Very successful campaigns were also carried out in Australia (36) and Italy (37, 38). For the prevention of onset DKA it seems essential that parents and caregivers such as teachers are familiar with the typical diabetes-related symptoms. According to an American study, 80% of the parents of children with T1D who were diagnosed without DKA had a specific suspicion of diabetes because they knew the symptoms, while the majority of the parents whose children had onset DKA neither knew about the disease nor the symptoms (39). Of course, it is also essential to teach medical students the symptoms and risk of onset DKA, as well as regularly refresh the relevant knowledge of practicing doctors in the context of further training.

Another approach to diagnose and treat children with T1D at an early stage is the establishment of screening programs, which are carried out very successfully, for example, in Bavaria. Among participants of the large-scale primary-care-based screening study Fr1da the prevalence of onset-DKA was extremely low (40), but at a population level there has not yet been a significant reduction in the onset-DKA prevalence, possibly due to the limited number of children participating in the screening program (18).

### Strengths and Limitations

The strengths of our study compared to others are that our setting is population-based with a high capture re-capture rate and an observation period of more than 30 years.

One limitation of our study is that data of migration status or fluency in German language are not collected in the Austrian incidence registry, so they could not be analyzed. However, the proportion of the population with migration background (41) in Austria has been rising steadily since 2008 (42) and was 24.4% in 2020 (43). It is not known if and how the migration wave of 2015 (44) contributed to the rise in DKA previous to the corona pandemic, but as migration status is an important risk factor for DKA at onset, this could be one possible explanation.

We further do not have information about the family income or socioeconomic status, duration of symptoms, number of health care contacts before diagnosis, and reason for a possible delay. We have no information about the duration of children's stay on intensive care units nor data on SARS-Cov2 antibodies in the newly diagnosed patients. However, our study was not planned as a COVID study and therefore the sub-analysis on the lockdown periods in 2020 should be understood as a side note.

## Conclusion

In summary, the high and increasing prevalence of DKA at onset of T1D in Austria is a serious problem, which was exacerbated by the COVID-19 pandemic. The problem of late and delayed T1D diagnosis will persist even after the COVID-19 crisis has been overcome if effective measures for DKA prevention are not taken. A lack of awareness of typical DKA or T1D symptoms in general population as well as in health care workers are suspected to be the reason for this alarming trend. Thus, strengthening diabetes awareness in caregivers, encouraging point-of-care diagnostics in primary health care, as well as a targeted approach for certain risk groups could contribute to tackle the problem.

## Data Availability Statement

The data analyzed in this study is subject to the following licenses/restrictions: the dataset is locally stored at the Medical University of Vienna, only for the purpose of the Austrian Diabetes incidence registry and Diabetes surveillance. The data set is available upon legitimated request. Requests to access these datasets should be directed to birgit.rami@meduniwien.ac.at.

## Author Contributions

KN was involved in data recruitment, conceptualized the study, interpreted the data, and wrote the manuscript. BR-M was the coordinator of the Austrian Diabetes Incidence Group, managed the data, and reviewed and edited the manuscript. TW performed the statistical analyses, generated the graphs, read, and edited the manuscript. MF, EF-R, CP, DM, and SH were involved in data recruitment, reviewed, and edited the manuscript. All authors approved the final manuscript as submitted and agreed to be accountable for all aspects of the work.

## Funding

The study was in part supported by Novo Nordisk Austria and Sanofi-Aventis Austria. The funders had no role in the study design, data collection and analysis, decision to publish, or preparation of the manuscript.

## Conflict of Interest

The authors declare that the research was conducted in the absence of any commercial or financial relationships that could be construed as a potential conflict of interest.

## Publisher's Note

All claims expressed in this article are solely those of the authors and do not necessarily represent those of their affiliated organizations, or those of the publisher, the editors and the reviewers. Any product that may be evaluated in this article, or claim that may be made by its manufacturer, is not guaranteed or endorsed by the publisher.

## Abbreviations

T1D: type 1 diabetes
DKA: diabetic ketoacidosis
APC: annual percent change
GP: general practitioners
COVID-19: corona-virus disease
SARS-CoV-2: Severe Acute Respiratory Syndrome Coronavirus-2.
