# Supplementary material for: Alarming Increase of Ketoacidosis Prevalence at Type 1 Diabetes-Onset in Austria—Results From a Nationwide Registry
Source: Front Pediatr. 2022 Feb 14;10:820156. doi: 10.3389/fped.2022.820156 (PMC8882618; doi:10.3389/fped.2022.820156)
Supplement: Supplementary file 1 [file Data_Sheet_1.PDF]

Supplement

Suppl. Table 1: Gender- and age specific prevalence (%) of DKA at T1D onset between 2012 and 2020

	Age at T1D onset (years)									
	0 - <2		2 - <5		5 - <10		10 - <15		total	
N (%)	female	male	female	male	female	male	female	male	female	male
no DKA	14 (27)	21 (28)	115 (58)	118 (58)	243 (57)	268 (65)	255 (59)	308 (53)	627 (57)	715 (56)
mild DKA	18 (35)	31 (42)	60 (30)	57 (28)	116 (27)	109 (26)	116 (27)	190 (32)	310 (28)	387 (30)
severe DKA	19 (37)	22 (30)	25 (13)	39 (15)	65 (15)	35 (9)	58 (14)	87 (15)	167 (15)	174 (14)
Total	51	74	200	205	424	412	429	585	1104	1276

Data are number of children and percentages.

COVID-19 Pandemic and lockdown 2020:

The effects of the COVID-19 pandemic in Austria resulted in three hard lockdown periods during 2020: March 16th - May 15th, November 17th - December 7th and December 26th 2020 - February 7th 2021 with many restrictions and limited access to medical care (14). During hard lockdown periods it was possible to leave the place of residence only for work, medical purposes, basic care, assistance, and exercise in the open air only. People were not allowed to meet anybody apart from other persons living in the same household. Access to medical care was limited, especially during the first lockdown period, to the extent that e.g., planned outpatient appointments were canceled or only held by telemedicine. All shops except those providing basic services (alimentary, etc.) restaurants, cultural, leisure and sports facilities were closed. Events were prohibited. School/university lessons were switched to home schooling and distance learning. Regular kindergarten was suspended. Parents were encouraged to send their children to external care (such as school or kindergarten) only in exceptional cases or in the absence of alternatives. Home office was encouraged. Religious communities forego worship services. During second hard lockdown exit restrictions came into force. During second and third lockdown it was allowed to meet one single person from a different household (14).

Appendix

Additional Austrian Diabetes Incidence Study Group Members:

Bauer M., Beran E., Bognar M., Bonfig W., Brugger M., Farid G., Glas K., Hassan J., Jäger A., Judmaier S., Kaderschabek N., Kitzler P., Klingbacher S., Kovacs U., Lindauer S., Lück U., Mayrhofer R., Neuhauser M., Niederseer R., Piringer G., Plank R., Polland V., Reichle D., Rojacher T., Schermann P., Schober J., Seick-Barbarini D., Seiwald M., Sickl E., Steigleder-Schweiger C., Wakolbinger G., Wutzl H., Zanier U. and Zimmerer F
